# Peritoneo‐cutaneous fistula from spilled gall bladder calculus following laparoscopic cholecystectomy

**DOI:** 10.1002/ccr3.903

**Published:** 2017-03-22

**Authors:** Suneed Kumar

**Affiliations:** ^1^ Department of General SurgeryGrant Govt. Medical College & Sir JJ Group of HospitalsMumbai400007MaharashtraIndia

**Keywords:** Complication, fistula, laparoscopic cholecystectomy, spilled gall stones, tract excision

## Abstract

Inadvertent spillage of gall stones is a rare yet important cause of delayed postcholecystectomy complications. Varied presentations and difficulty in diagnosis are the hallmarks, making it crucial to have a high index of suspicion to detect and intervene appropriately. Peritoneo‐cutaneous fistulae from the retained stone can be completely excised in toto.

Question: A 54‐yr‐old male presented with persistent sero‐purulent discharge from sinus at right lateral port site nearly 8 months after laparoscopic cholecystectomy. He was of generally good health, with no fever or weight loss, good appetite, and bowel function. By contrast, cross‐sectional imaging with sinogram showed an 8‐cm long tract, with minimal collection but no obvious mass. The procedure performed involved coring out of entire tract with contained material. In view of the images provided, what may be the most likely diagnosis?

- Intra‐abdominal abscess
- Spilled gall stones
- Biliary fistula
- Entero‐ or Colo‐cutaneous fistula

Answer: B) Spilled gall stones.

## Introduction

Spillage of gall stones following conventional and single incision laparoscopic procedures is on the rise due to the increasing numbers of difficult cases performed, namely elderly age group, obese patient, acute inflammation, and adhesions. Literature reports an overall incidence of spilled stones as 5–40%, but causing morbidity in only about 0.08–0.3% of cases 1. While majority of these cases present as intra‐abdominal abscess requiring drainage, only few present with persistent discharging peritoneo‐cutaneous fistula.

## Case Report

**Figure 1:**
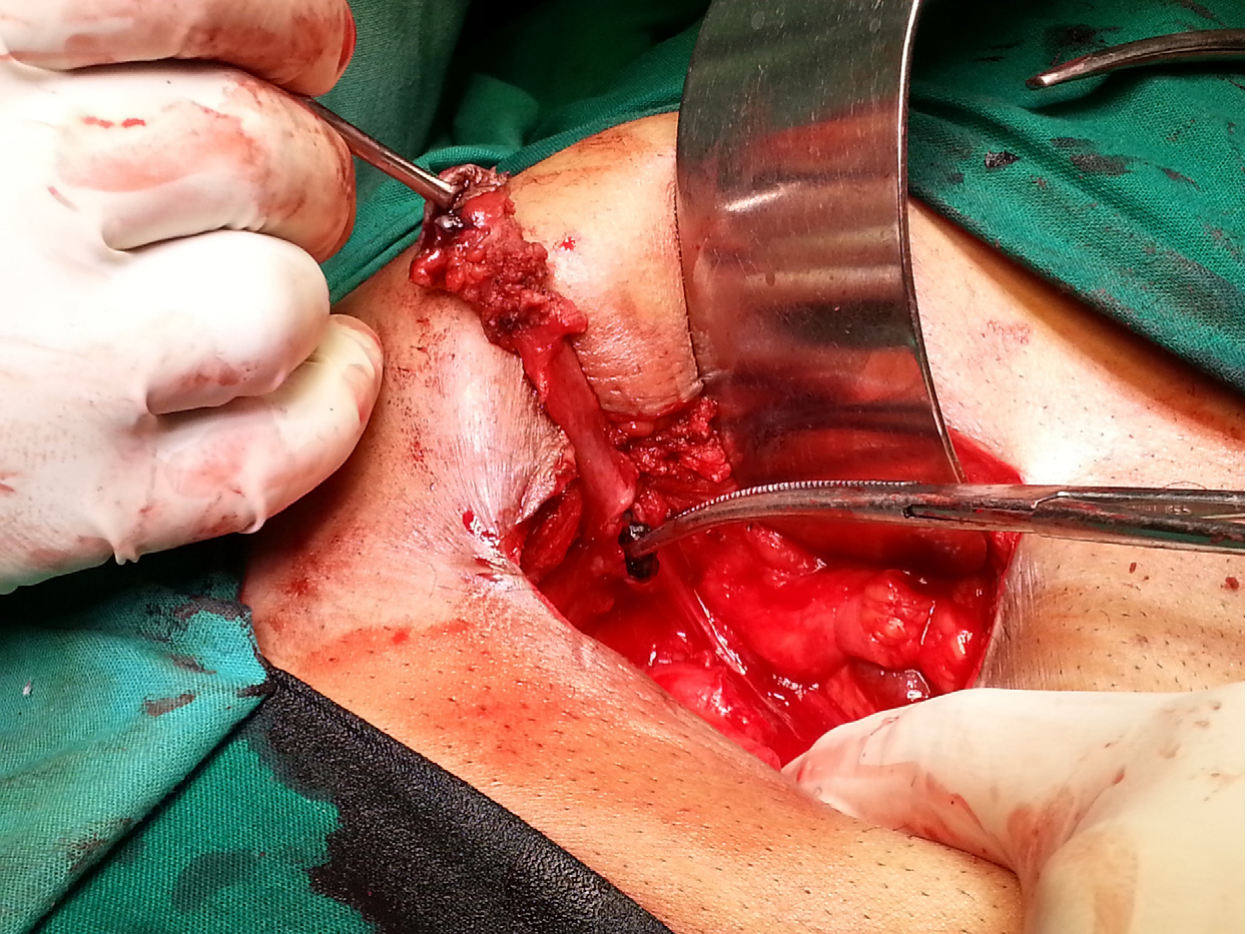
Intraoperative photograph of excised fistula tract terminating in retained spilled gall stone.

**Figure 2:**
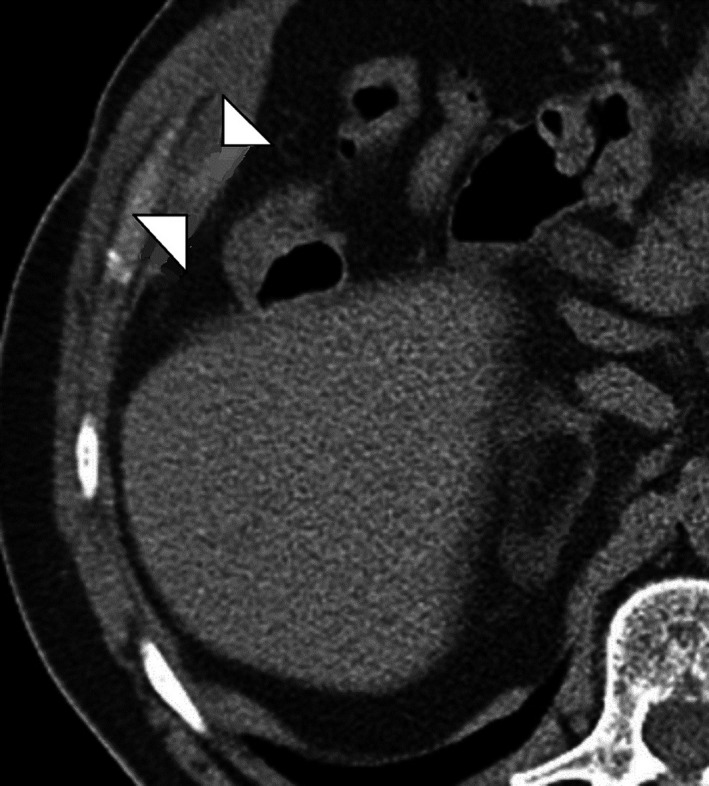
Axial section of contrast enhanced CT scan, showing evidence of small walled off collection within the subhepatic peritoneal cavity, marked between the two arrow heads.

**Figure 3:**
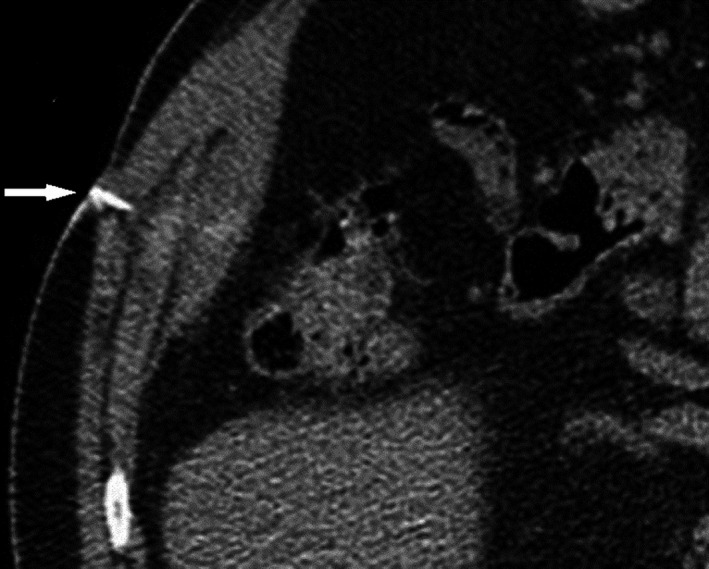
Axial section of contrast enhanced CT, with sinogram probe (arrow) within the tract, and no evidence of filling defect or side‐branching of tract.

The patient underwent conventional laparoscopic cholecystectomy at a district level hospital 8 months prior to presentation. He complained of initial serous, followed by intermittent purulent discharge from the right midclavicular working port site. He was otherwise healthy, with no previous comorbid illness, or associated symptoms. Superficial probe ultrasound showed no parietal wall collections, with no obvious detectable intra‐abdominal pathology. Contrast enhanced computed tomography (CT) with sinogram subsequently showed evidence of an 8‐cm‐long tract extending from skin to the subhepatic region without evidence of any stone or mass at the end (Figures 2 and 3). Under combined spinal–epidural anesthesia, wide local excision of the tract around an elliptical skin incision was done, with findings of an 8.5‐cm‐long fistula tract ending in the subhepatic peritoneal cavity at a walled off retained spilled gall stone measuring 2 × 1.5 cm, with minimal sero‐purulent collection. Entire tract with gall stone was excised in toto (Figure 1). Histopathological assessment of the fistula tract revealed only the evidence of chronic inflammation. The patient was followed up every 6 months for 2 years with clinical examination and ultrasound, and was asymptomatic at last visit.

## Discussion and Literature Review

Spillage of gall stones can occur either during dissection or at specimen retrieval phase in cholecystectomy 2. Such spillage are usually detected instantly, where in the ideal approach would be laparoscopic stone removal with thorough lavage. Need for conversion to open procedure for spilled stones is debatable 1, since the incidence of complications arising from this is extremely low. Otherwise, spillage of stones may go undetected and present early in the first few days or weeks following surgery with intra‐abdominal abscesses, in the background of systemic sepsis. This will require drainage of abscess along with intravenous antibiotics, hydration, and supportive care. The third and rarest presentation is late, as in our case, beyond 6 months, with the development of fistulae, persistent discharging sinuses, distant migration of stones and delayed abscesses, intestinal obstruction, urinary symptoms, etc.

Intra‐abdominal abscess is the commonest reported complication, with a mean incidence of 0.1–2.9% in patients with spilled stones. This may present anytime between 10 days and 72 months post index surgery 3. Whereas percutaneous therapy may be sufficient for a selected few cases, majority (86–90.5%) cases will require open surgery, abscess drainage, and stone extraction 3. The next commonest complication of spilled gall stones is wound infections and parietal abscess with chronic discharging sinuses. Fistulization usually occurs as colo‐cutaneous or biliary‐cutaneous fistulae 1, 4.

Management of fistulae such as the peritoneo‐cutaneous fistula in our case includes diagnosis via computed tomography or magnetic resonance cross‐sectional imaging and sinogram, to identify the length and course of tract, its extent and termination; and identify any associated anatomical disturbances. When associated with intra‐abdominal abscess, local exploration and drainage may be worthwhile, but definitive treatment is by complete tract excision along with the culprit stone, thorough lavage, and primary or delayed closure, depending on extent of purulent collection. Needless to say, prophylaxis still remains the forefront, with meticulous dissection, specimen retrieval, and final thorough inspection prior to closure.

Option (A), intra‐abdominal abscess would cause systemic symptoms with abdominal pain. Its management would initially involve percutaneous drainage. Option (C), biliary fistula would usually present with icterus, general ill‐health, raised liver markers, and show dilated intrahepatic biliary radicles on imaging. Management would involve invasive or noninvasive cholangio‐pancreatography, which would further decide course of treatment. Option (D), entero‐ or colo‐cutaneous fistula is quite a morbid condition, with patient showing systemic symptoms, deranged nutritional status and sarcopenia. Hence, in this case, best answer is Option (B), spilled gall stones (Figs 1, 2, 3).

## Conclusion

Peritoneo‐cutaneous fistula is a rare but important presentation of spilled gall stones following laparoscopic cholecystectomy. There is thus need to suspect this and manage accordingly, especially in cases presenting more than 6 months following index procedure. Wide excision of fistula tract along with culprit stone and along with thorough lavage is the treatment of choice.

## Authorship

SK: Member of the primary treating surgical team. Preparation of manuscript, study design, and proof reading. Photographs taken and edited as per requirements. Literature review and discussions section.

## Conflict of Interests

The author hereby declares no conflict of interests
